# Solitary Pulmonary Capillary Hemangioma: CT and PET-CT Features with Clinicopathologic Correlation

**DOI:** 10.3390/diagnostics12112618

**Published:** 2022-10-28

**Authors:** Min Ju Kim, Wonju Hong, Tae Jung Kim, Joungho Han, Yoon-La Choi, Joon Young Choi, Sang Min Lee, Sung Ho Hwang

**Affiliations:** 1 Department of Radiology, Samsung Medical Center, Sungkyunkwan University School of Medicine, 81 Irwon-ro, Gangnam-gu, Seoul 06351, Korea; 2 Department of Radiology, Seoul National University Hospital, 101 Daehak-ro, Jongno-gu, Seoul 03080, Korea; 3 Department of Radiology, Hallym University Sacred Heart Hospital, 22 Gwanpyeong-ro 170beon-gil, Dongan-gu, Anyang 14068, Korea; 4 Department of Pathology, Samsung Medical Center, Sungkyunkwan University School of Medicine, 81 Irwon-ro, Gangnam-gu, Seoul 06351, Korea; 5 Department of Nuclear Medicine, Samsung Medical Center, Sungkyunkwan University School of Medicine, 81 Irwon-ro, Gangnam-gu, Seoul 06351, Korea; 6 Department of Radiology, Asan Medical Center, University of Ulsan College of Medicine, 88 Olympic-ro 43-gil, Songpa-gu, Seoul 05505, Korea; 7 Department of Radiology, Korea University Anam Hospital, Korea University Medicine, 73 Goryeodae-ro, Seongbuk-gu, Seoul 02841, Korea

**Keywords:** solitary pulmonary nodule, hemangioma, capillary, adenocarcinoma of lung, lung neoplasms

## Abstract

The aim of this study was to evaluate the CT and PET-CT features of solitary pulmonary capillary hemangioma (SPCH) with clinicopathologic correlations. This retrospective study included 17 patients with histologically proven SPCH from four tertiary institutions. The clinical, pathological and imaging findings of SPCH were reviewed. The CT features assessed included lesion location, size, density, contour, margin, enhancement, presence of air bronchogram, perivascular lucency and pleural retraction, and ^18^F-fluorodeoxyglucose uptake on PET-CT. Changes in the size during the follow-up period were also evaluated. Imaging features were correlated with the clinicopathologic findings. The mean age of the patients was 47 years (range 30–60 years). All SPCHs were incidentally detected during screening CT examinations (*n* = 13, 76%) or during cancer work-up (*n* = 4, 24%). Most SPCHs appeared as part-solid nodules (*n* = 15, 88%), the remaining appeared as a pure ground-glass nodule or a pure solid nodule, respectively. Most had smooth contours (*n* = 16, 94%), while one had a lobulated contour. Nine SPCHs (53%) showed ill-defined margins. Air bronchogram was present in ten (59%) SPCHs, and perivascular lucency in two (12%). All SPCHs exhibited hypoattenuation on contrast-enhanced CT and hypometabolism on PET-CT. During the follow-up period (mean 14.8 ± 17.7 months), the lesions showed no change in size or density in ten SPCHs (59%), decreased or fluctuation in size and density in three (18%). SPCH is often incidentally detected in young and middle-aged adults, commonly as an ill-defined part-solid nodule that may accompany air bronchogram, perivascular lucency, and fluctuation in size or density on CT and hypometabolism on PET-CT.

## 1. Introduction

Solitary pulmonary capillary hemangioma (SPCH) is a benign tumor of capillary vessels and is characterized by the proliferation of capillaries in the alveolar septa [1,2,3,4]. SPCH was first described in the literature as “pulmonary capillary hemangiomatosis-like foci” in 2000, in a report of eight autopsies [2]. Unlike pulmonary capillary hemangiomatosis, which is closely related to pulmonary hypertension and poor prognosis [5,6,7,8], SPCHs often present as an incidental solitary pulmonary nodule with excellent prognosis [1,3,4,9,10,11,12,13,14,15].

There have been several reports about the radiologic findings of SPCH [4,10,11,12,13,14,15]. The main CT finding of SPCH reported so far is a solitary part-solid or ground-glass nodule. Therefore, although SPCH is a benign lesion, it is often surgically resected because it mimics lung adenocarcinoma due to its ground-glass or part-solid appearance on CT. However, in these studies, SPCH was merely described as a subsolid nodule by focusing only on nodule attenuation [4,10,11,12,13,14,15], and detailed imaging features have not been described. A recent preliminary study tried to distinguish SPCH from lung adenocarcinoma with radiomic features, but the study has limitations due to its small sample size [16].

The detection of subsolid nodules is increasing due to the widespread use of CT, and diagnosis and management of subsolid nodules is a major concern in the era of lung cancer screening. Radiologic features that can differentiate between lung adenocarcinoma and SPCH may be helpful in the proper management of subsolid nodules. Therefore, this study aimed to evaluate the CT and PET-CT features of SPCH with clinicopathologic correlations.

## 2. Methods

### 2.1. Patients

This retrospective study was approved by the institutional review board of each hospital, and the requirement for informed consent was waived. The analysis was based on patient data collected from four tertiary referral centers in South Korea: Samsung Medical Center, Seoul National University Hospital, Asan Medical Center, and Korea University Anam Hospital. A search of the electronic medical record database was performed using the search terms “pulmonary capillary hemangioma” to identify patients who underwent surgical resection or biopsy of SPCH between January 2008 and September 2020. The inclusion criteria were patients with a histologically confirmed diagnosis of SPCH and available preoperative or pre-procedural CT. The exclusion criteria were patients with pulmonary lesions smaller than 5 mm, which were too small to be characterized on CT. One patient with a small lesion size (3 mm) was excluded, and 17 patients in total were included. The collected demographic parameters and clinical details included sex, age, smoking history, and underlying medical conditions and pulmonary diseases (Table 1).

### 2.2. CT Imaging Analysis

CT examinations were performed in multiple centers using helical CT scanners. All patients underwent CT scans from the lung apex to the level of the middle portion of both kidneys at the suspended maximum inspiration. Scans were performed at 120 kVp with mAs ranging from 30 to 200 mAs with or without automatic exposure control according to the capability of each scanner. The image data were reconstructed with a section thickness of 1.0–1.25 mm using a sharp algorithm and 2.5–5.0 mm using a smooth algorithm. Post-contrast CT images were obtained in 15 patients between 50 and 70 s after starting the injection of 100–120 mL of nonionic contrast medium. CT examinations were retrospectively and independently reviewed by two radiologists who specialize in thoracic imaging (W.H. and S.M.L. with 6 and 16 years of experience in thoracic imaging, respectively). Consensus was reached after a discussion was conducted between the two readers for all the images, and in case of disagreement, a third senior radiologist specializing in thoracic imaging (T.J.K. with 23 years of experience) made the final decision. Each reader was asked to assess the lesion location (central or peripheral), size, maximum diameter of the solid component, percentage of the solid component, density (solid, part-solid, or ground-glass), contour (smooth, lobulated, spiculated), margin (well-defined or ill-defined), enhancement, presence of air bronchogram, perivascular lucency and pleural retraction and fluorine-18-Fluorodeoxyglucose (FDG) uptake on PET-CT [17]. Tumors were categorized as central or peripheral by location within one-third or two-thirds of the hemithorax [18]. Changes in the size or density during the follow-up period were also evaluated. The percentage of the solid component was calculated as follows: [maximum diameter of the solid component/maximum diameter of the whole lesion] × 100, where the maximum diameter of the whole lesion included both ground-glass opacity and solid components. Enhancement was described by its attenuation relative to the chest wall muscles on contrast-enhanced CT; Hounsfield units (HU) were measured in solid portions of the lesions, and HU was not measured for lesions that had unreliable HU due to partial volume with air or air bronchogram. Ten of the seventeen patients also underwent ^18^F-FDG PET-CT. The maximum standardized uptake value (SUV_max_) was measured. If SUV_max_ was not reliably measured or unavailable, FDG uptake was qualitatively evaluated as either hypometabolic or hypermetabolic compared with the mediastinum.

### 2.3. Pathology Analysis

Histologic diagnoses of SPCH were made by surgical resection (*n* = 15) or percutaneous core needle biopsy (*n* = 2) (Table 2). One experienced pathologist (J.H. with 25 years of experience) interpreted all tissue sections. SPCH was diagnosed based on pathologic features such as the proliferation of capillaries in the alveolar septa, and the vascular nature of SPCH was examined via immunohistochemistry using the vascular marker CD31. In 15 patients who underwent surgical excision, we examined each radiologic finding that correlated with the pathologic specimens. Correlations between radiologic and pathologic findings were made by focusing on the morphologic CT features such as marginal characteristics (e.g., ill-defined and well-defined), nodule attenuation (e.g., ground-glass, part-solid, and solid), and internal characteristics of the nodule (e.g., air bronchogram and perivascular lucency). This was performed by a radiologist (T.J.K.) and pathologist (J.H.), and decisions were reached by consensus.

## 3. Results

### 3.1. Demographics and Follow-Up Results

The demographic and clinical findings of the 17 patients with SPCH are summarized in Table 1. All 17 patients were asymptomatic, and lesions were detected incidentally during routine health check-ups (*n* = 13, 76%) or cancer work-up (*n* = 4, 24%). The age range was 30–60 years, with a mean age of 47 years. Eleven patients (65%) had never smoked, and fourteen patients (82%) did not have any pulmonary disease. None of the patients had emphysema, interstitial lung abnormalities, or pulmonary fibrosis. After surgical resection of the SPCH lesions (*n* = 15), fourteen patients demonstrated no evidence of recurrent disease during the follow-up period (mean 30.7 ± 32.2 months, range 0.5–106.6 months). In one patient with an SPCH lesion that was incompletely resected, the remnant lesion did not show any change during the follow-up period (64.2 months). In two patients who did not undergo surgical resection, one lesion decreased in density, and the other lesion did not change in size or density during the follow-up period (12.6 months and 9.1 months, respectively).

### 3.2. CT and PET-CT Features

**Figure 1:**
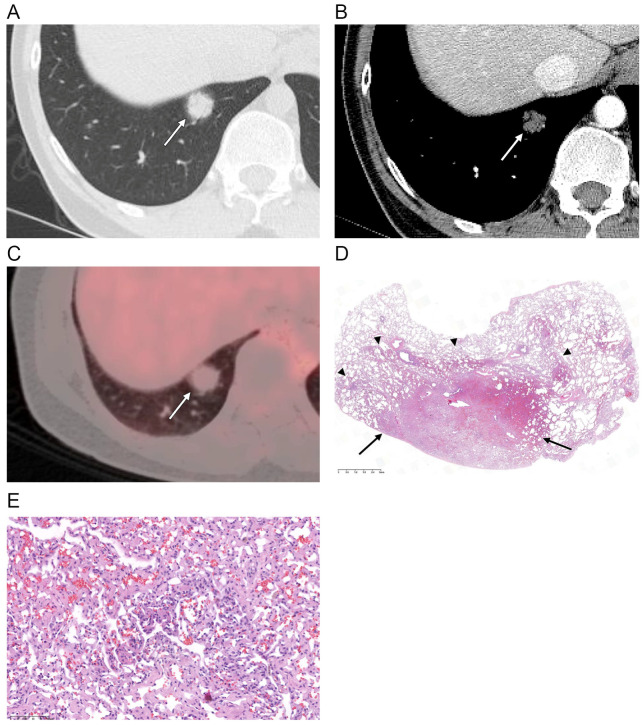
(**A**) Axial CT scan (section thickness, 1.25 mm) obtained in a 37-year-old male shows a part-solid nodule (arrow) in the right lower lobe. Central solid portion with ill-defined peripheral ground-glass opacity is demonstrated. (**B**) Post-contrast CT image shows hypoattenuation of the nodule (arrow) compared with the chest wall muscles. (**C**) PET-CT scan shows a hypometabolic nodule (arrow) with a SUV_max_ of 1.7 (**D**,**E**) Photomicrographs (original magnification, ×10, and ×200; hematoxylin–eosin [H & E] stain) show the central portion of the solitary pulmonary capillary hemangioma congested with more thickened and proliferated capillary vessels with irregular anastomosis (arrows), and the peripheral portion made of proliferated capillary vessels mixed with normal lung parenchyma (arrowheads).

**Figure 2:**
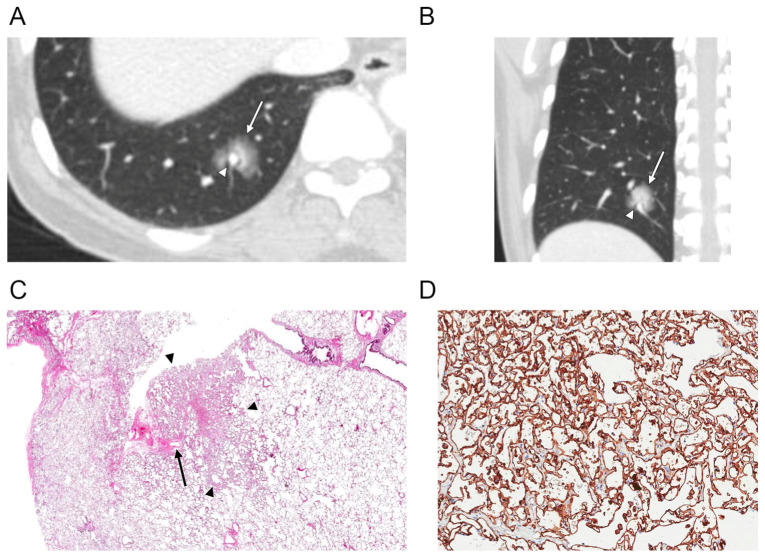
(**A**,**B**) Axial and coronal CT scans of the right lung (section thickness, 2.5 mm) in a 52-year-old female demonstrate a part-solid nodule (arrow) in the right lower lobe. There is a perivascular lucency (arrowhead) around the pulmonary vein that courses across the nodule. (**C**) Photomicrograph (original magnification, ×4; H & E stain) shows a solitary pulmonary capillary hemangioma (arrowheads) with dilatation and edematous congestion of the pulmonary veins, causing thickening of the interlobular septa. In these areas of vascular congestion within the interlobular septa, there was relative sparing of capillary proliferation (arrow). (**D**) CD31 immunohistochemical stain highlights the endothelial tumor cells (1:80, Dako, Glostrup, Denmark, original magnification, ×200).

The CT features of the SPCH are summarized in Table 3. Fifteen SPCHs (88%) appeared as part-solid nodules, while the remaining two lesions appeared as either ground-glass nodule or solid nodule. Most part-solid nodules (13 of 15, 87%) had a ground-glass halo surrounding the central solid portion (Figure 1A and Figure 2A). The remaining two part-solid nodules (13%) had ground-glass opacity mixed with the area of the solid portion. The mean total diameter of the SPCH was 13.4 ± 4.2 mm, with the mean diameter of the solid portion measuring 9.1 ± 4.7 mm. The mean percentage of the solid component measured was 67.5 ± 28.2%. The lesions were mainly located in the right lower lobe (*n* = 8, 48%) and the left lower lobe (*n* = 5, 30%). Fifteen SPCHs (88%) were found in the peripheral lung zones. Most SPCHs (*n* = 16, 94%) had smooth contours (Figure 1 and Figure 2), except for one that exhibited lobulated contours. None of the lesions had spiculated or irregular contours. Eight SPCHs (47%) had well-defined margins, whereas nine SPCHs (53%) had ill-defined margins with a fuzzy or blurred tumor-lung interface appearing as a lunar halo (Figure 1A and Figure 2A). Ten SPCHs (59%) demonstrated an air bronchogram (Figure 1A). Two SPCHs demonstrated perivascular lucency around the pulmonary veins traversing the lesions (Figure 2A,B). Post-contrast CT scans were available in 15 patients. Fourteen SPCHs were hypoattenuating (Figure 1B), and one SPCH was iso-attenuating compared with the chest wall muscles on post-contrast CT images. SPCHs of all ten patients with PET-CT scans available were hypometabolic, with SUV_max_ ranging from 0.8 to 1.73 in four SPCHs in which SUV was reliably measured (Figure 1C). The mean size of SPCHs with PET-CT scans was 14 mm and most appeared as part-solid nodules (*n* = 9), except for one that appeared as a solid nodule. All cases in our study were initially thought to be primary lung adenocarcinomas.

**Figure 3:**
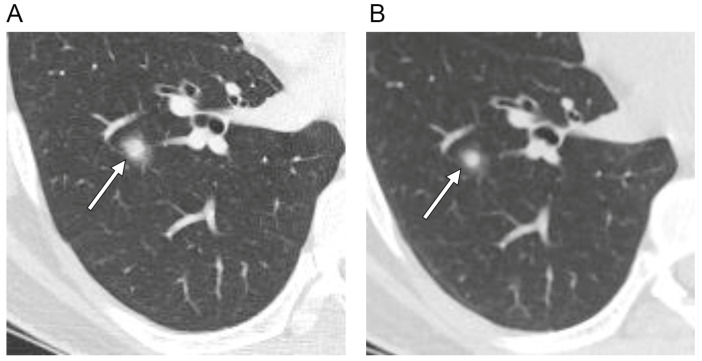

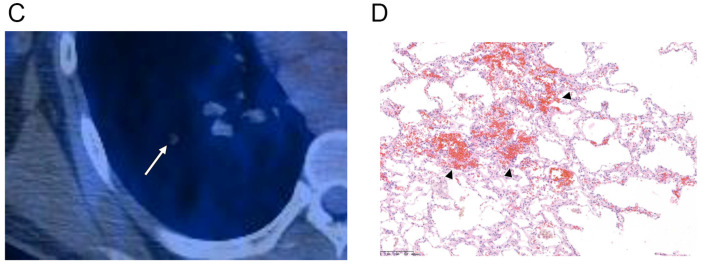
Axial CT scans of the right lung (section thickness, 2.5 mm) in a 36-year-old male with a solitary pulmonary capillary hemangioma demonstrate: (**A**) part-solid nodule with ill-defined margin (arrow) (**B**) that decreased in size and density during the three-month follow-up. (**C**) Magnified PET-CT image shows a hypometabolic nodule (arrow). (**D**) Photomicrograph (original magnification, ×100; H & E stain) shows densely proliferating and dilated capillaries within the alveolar septa. Additionally, note the red blood cell collections (arrowheads) within the dilated capillaries that may be the possible mechanism of change in size and density on follow-up CT scan.

**Figure 4:**
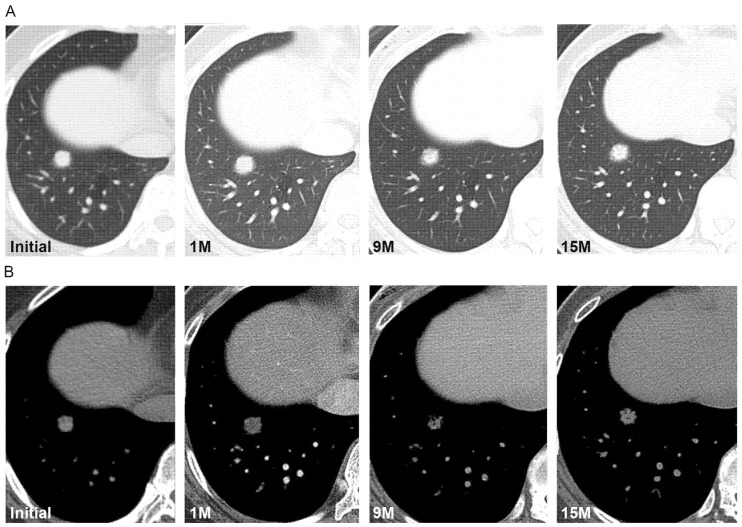
(**A**,**B**) Axial CT scans of the right lung in a 52-year-old female demonstrate a part-solid nodule that fluctuates in size and density during the 15-month follow-up. Initial CT scan demonstrates a part-solid nodule in the right lower lobe with a diameter of 14 mm. One month follow-up CT scan demonstrates the increased size of the nodule (16 mm). Nine-month follow-up CT scan demonstrates the decreased density and size (14 mm) of the nodule; fifteen-month follow-up CT scan demonstrates the increased density but no change in the nodule size. Surgical biopsy results confirmed the diagnosis of solitary pulmonary capillary hemangioma.

Follow-up CT scans before histologic diagnosis were available in all 17 patients. The mean follow-up period was 14.8 ± 17.7 months (range, 0.7–64.9 months). During the follow-up (mean 7.2 ± 6.5 months), ten SPCHs (59%) did not show changes in size, shape, or density. One SPCH (6%) showed a decrease in density (Figure 3), and two (12%) showed changes in shape or fluctuation in size during follow-up (mean 8.8 ± 4.9 months) (Figure 4). Four SPCHs (24%) showed an increase in size during the follow-up period (38.2 ± 20.1 months).

### 3.3. Histopathologic Findings and Correlation with CT Features

Histologically, SPCHs consist of thickened alveolar septa with the proliferation of anastomosing vascular spaces, replacing the normal lung parenchymal tissue (Figure 1D,E). The central portion of the SPCH was especially congested with more thickened and proliferated capillary vessels and had irregular anastomosis, which correlated with the higher density and solid portion of the lesion on CT (Figure 1). The peripheral portion of the SPCH had tissue made of proliferated capillary vessels mixed with normal lung parenchyma, and the borders between the two were ill-defined, which correlated with ground-glass opacity at the peripheral portion of the lesion and ill-defined margins on CT (Figure 1).

There was also variable dilatation and edematous congestion of the pulmonary veins, causing thickening of the interlobular septa. In these areas of vascular congestion within the interlobular septa, there was relative sparing of capillary proliferation (Figure 2C), which correlated with perivascular lucency around the pulmonary vein that traverse the SPCH (Figure 2A,B).

## 4. Discussion

Based on our study, SPCH can be described as an incidentally detected pulmonary lesion in young and middle-aged adults, commonly presenting as an ill-defined part-solid nodule that may accompany air bronchogram, perivascular lucency, and fluctuation in size or density on CT and hypometabolism on PET-CT.

SPCH is a benign tumor consisting of proliferated capillaries in the alveolar septa [1,2,3,4], which presents as a solitary nodule on CT. Several case reports and small case series have characterized SPCH based on its density on CT, describing it as a part-solid or ground-glass nodule [4,10,11,12,13,14,15]. However, detailed imaging features of SPCH, especially those with pathological correlations, have been scarcely described in the literature. Although SPCH is a benign lesion, it is commonly mistaken for lung adenocarcinoma due to its subsolid appearance on CT. Indeed, the initial radiologic diagnoses of all SPCHs in our study were lung adenocarcinomas. The clinical value of distinguishing SPCH from lung adenocarcinoma has also been recognized by a recent preliminary report that introduced a discriminant model based on radiomics features [16]. However, this study is limited in that the radiomic features were extracted from only 13 cases of SPCH [16]. To the best of our knowledge, our study is the first to provide detailed analyses of the imaging features of SPCH with pathologic correlation. According to our study, SPCH may differ from lung adenocarcinoma with several imaging features, such as ill-defined margins, perivascular lucency, and fluctuations in size and density on CT, and hypometabolism on PET-CT. Despite such differences in imaging features, biopsy or surgical excision of these pulmonary lesions is inevitable for a definitive diagnosis. However, when patients present with pulmonary lesions that show these imaging features that favor SPCH as described in our study, we expect that our study findings may be useful in treatment planning and when deciding the surgical extent (e.g., limited surgery vs. standard lobectomy). Furthermore, our findings may also be helpful in managing patients who are not clinically fit for surgery in more conservative ways, such as with imaging surveillance.

Interestingly, more than half of the SPCHs in our study had ill-defined margins, which correlated with the histological findings. The denser central portion of SPCH was correlated with more congested and extensive proliferation of irregularly anastomosing capillaries that replace the normal lung tissue histologically. Although red blood cells are not often preserved in the specimen slides, we suspect that red blood cells within the capillaries may also play an important role in the increased opacity in the central portions of the SPCH. In contrast, the peripheral portions of the SPCHs were mixed with normal lung parenchyma. The borders between the normal lung parenchyma and tissue with proliferated capillaries were difficult to distinguish in the periphery of the lesions, contributing to the peripheral ground-glass opacity and ill-defined margins of the SPCH.

Some of the SPCHs in our study showed perivascular lucency around the pulmonary vein that traverses across the lesion. Microscopically, the pulmonary veins also showed dilatation and edematous congestion within the interlobular septa. In these areas, capillary proliferation was relatively spared, possibly explaining the lucency around the pulmonary vein that courses through the lesion. This perivascular lucency was observed in SPCH developing over two adjacent secondary pulmonary lobules, which has not been described in lung adenocarcinoma with a lepidic growth pattern [19,20,21,22].

In our study, most SPCHs were hypoattenuating to the chest wall muscles. This is unlike other vascular tumors or tumors with angiomatous components, such as cavernous hemangiomas and sclerosing pneumocytomas, which frequently show prominent contrast enhancement [23,24]. Such poor contrast enhancement of SPCH may be explained by the less prominent vascular flow within the capillary network in SPCH compared with that within larger vascular structures found in other hypervascular tumors.

In our study, all SPCHs showed hypometabolism, and the SUV_max_ ranged from 0.8 to 1.7, which are substantially lower than the FDG uptake of lung adenocarcinomas manifesting as part-solid lesions [25]. This scanty FDG uptake may also help differentiate SPCH from part-solid lung adenocarcinomas.

In our study, most SPCHs did not change in size, shape, or density during the follow-up period. This included a part-solid nodule with a solid portion measuring 16 mm in diameter, which did not change in size during the follow-up period of 18 months. This is another distinguishing characteristic of SPCH, considering the typical doubling time of lung cancer [26,27,28]. Interestingly, one SPCH showed a decrease in density, and two showed changes in shape or fluctuation in size during follow-up. Matsushita et al. reported an SPCH that decreased in density when CT was performed in the prone position for CT-guided needle biopsy [13]. Similar to this finding, the changes in density, size, and shape of several SPCH cases in our study may be explained by changes in the vascular pool within the capillaries, as well as by changes in the extent and stage of intralesional hemorrhage, which may be associated with SPCH histologically [4,13,24].

There are several limitations to our study. First, our study was retrospective in nature and had a small number of patients. Nevertheless, our study is one of the largest case series through case collection from four tertiary referral centers in South Korea. Second, because this was a multi-institutional study, CT scanners and CT protocols were inconsistent. However, thin-section CT images were available for the analysis of the detailed features of SPCHs.

In conclusion, our study suggests that SPCH can be characterized as an incidentally detected pulmonary lesion in young and middle-aged adults, commonly presenting as an ill-defined, part-solid nodule that may accompany air bronchogram, perivascular lucency, and fluctuation in size or density on CT and hypometabolism on PET-CT.

## Figures and Tables

**Table 1:** Demographics of 17 patients with histologically proven solitary pulmonary capillary hemangioma.

Variables	Data (*n* = 17)
Age (y)	47 (30–60) *
Female	10 (59)
Smoking history	6 (35)
Medical history	
Diabetes mellitus	1 (6)
Hypertension	1 (6)
Malignancy **	3 (18)
Lung cancer	1 (6)
Tuberculosis or NTM disease	2 (12)
Emphysema	0 (0)
Interstitial lung abnormality ^†^	0 (0)

Unless otherwise indicated, data are the number of patients with percentages in parentheses. * Data is the median, with the range in parentheses. ** Malignancies include breast cancer, colon cancer, and lymphoma. ^†^ Interstitial lung abnormalities include ground-glass opacity, reticulation, honeycombing, and non-emphysematous cysts. NTM, Non-tuberculous mycobacteria.

**Table 2:** Mode of detection, treatment done, and current disease status of 17 patients with histologically proved solitary pulmonary capillary hemangioma.

Variables	Data (*n* = 17)
Mode of detection	
Screening	13 (76)
Cancer work-up	4 (24)
Mode of histologic confirmation	
Core needle biopsy	2 (12)
Surgical resection	15 (88)
Current disease status	
No evidence of disease	14 (82)
No interval change	2 (12) *
Decrease in size/density	1 (6) **

Data are the number of patients with percentages in parentheses. * Corresponds to one lesion that was incompletely resected and the other lesion that only underwent biopsy. ** Corresponds to one lesion that only underwent biopsy.

**Table 3:** CT and PET-CT findings of solitary pulmonary capillary hemangioma.

Variables	All Patients (*n* = 17)
Max. diameter of the whole lesion (mm)	13.4 ± 4.2
Max. diameter of the solid component (mm)	9.1 ± 4.7
Percentage of the solid component (%)	67.5 ± 28.2
Nodule density	
Ground-glass	1 (6)
Part-solid	15 (88)
Solid	1 (6)
Lobe	
RUL/RML/RLL	1 (6)/1 (6)/8 (48)
LUL/LLL	2 (12)/5 (30)
Location	
Central	2 (12)
Peripheral	15 (88)
Contour	
Smooth	16 (94)
Lobulated	1 (6)
Margin	
Well-defined	8 (47)
Ill-defined	9 (53)
Perivascular lucency	2 (12)
Air bronchogram	10 (59)
Pleural retraction *	0 (0)
Enhancement **	
Hypoattenuation	14 (93)
Isoattenuation	1 (7)
FDG uptake on PET-CT ^†^	
Hypometabolic	10 (100)
Change during follow-up period	
None	10 (59)
Decrease in size/density or fluctuation in size/shape/density	3 (18)
Increase in size/density	4 (24)

Values are given as the mean ± standard deviation or number of patients with percentages in parentheses. * Eight out of 17 patients had nodules that were juxtapleural in location, and the percentage represents the percentage of the total number of patients with juxtapleural nodules. ** Contrast-enhanced CT was available in 15 out of 17 patients. Attenuation on contrast-enhanced CT was compared with attenuation of the chest wall muscle. ^†^ PET-CT scans were available in ten out of 17 patients, and the percentage represents the percentage of the total number of patients with available PET-CT. FDG uptake was compared with that of the mediastinum.

## Data Availability

The data presented in this study are available on request from the corresponding author.

## Abbreviations

SPCH	Solitary pulmonary capillary hemangioma
SUV_max_	Maximum standardized uptake value
